# In silico analysis of AHJD-like viruses, *Staphylococcus aureus* phages S24-1 and S13′, and study of phage S24-1 adsorption

**DOI:** 10.1002/mbo3.166

**Published:** 2014-03-04

**Authors:** Jumpei Uchiyama, Iyo Takemura-Uchiyama, Shin-ichiro Kato, Miho Sato, Takako Ujihara, Hidehito Matsui, Hideaki Hanaki, Masanori Daibata, Shigenobu Matsuzaki

**Affiliations:** 1 Department of Microbiology and Infection, Faculty of Medicine, Kochi UniversityNankoku City, Kochi, Japan; 2 Center for Innovative and Translational Medicine, Faculty of Medicine, Kochi UniversityNankoku City, Kochi, Japan; 3 Research Institute of Molecular Genetics, Kochi UniversityNankoku City, Kochi, Japan; 4 Science Research Center, Kochi UniversityNankoku City, Kochi, Japan; 5 Research Center for Infections and Antimicrobials, Kitasato Institute for Life Sciences, Kitasato UniversityTokyo, Japan

**Keywords:** Bacteriophage, phage adsorption, *Staphylococcus aureus*, wall teichoic acid

## Abstract

*Staphylococcus aureus* is a clinically important bacterium that is commensal in both humans and animals. Bacteriophage (phage) attachment to the host bacterial surface is an important process during phage infection, which involves interactions between phage receptor-binding proteins and host receptor molecules. However, little information is available on the receptor-binding protein of *S. aureus* phages. *S. aureus* virulent phages S24-1 and S13′ (family *Podoviridae*, genus AHJD-like viruses) were isolated from sewage. In the present study, we investigated the receptor-binding protein of AHJD-like viruses using phage S24-1. First, based on a comparative genomic analysis of phages S24-1 and S13′, open reading frame 16 (ORF16) of phage S24-1 was speculated to be the receptor-binding protein, which possibly determines the host range. Second, we demonstrated that this was the receptor-binding protein of phage S24-1. Third, our study suggested that wall teichoic acids in the cell walls of *S. aureus* are the main receptor molecules for ORF16 and phage S24-1. Finally, the C-terminal region of ORF16 may be essential for binding to *S. aureus*. These results strongly suggest that ORF16 of phage S24-1 and its homologs may be the receptor-binding proteins of AHJD-like viruses.

## Introduction

*Staphylococcus aureus* is a Gram-positive coccus, and an opportunistic pathogen, but a commensal bacterium in humans and various animals, and it is also present in the environment (Gabutti et al. 2000; De Vos et al. 2009; Graveland et al. 2011; Goodwin et al. 2012). Methicillin-resistant *S. aureus* (MRSA) is prevalent in both humans and animals, and has caused serious infections in clinical settings in many countries (Grundmann et al. 2006; Chambers and Deleo 2009; Graveland et al. 2011).

Bacterial viruses, bacteriophages (phages), are ubiquitous in the environment and are the most diverse life forms on earth (Hendrix et al. 1999). Approximately 96% of the isolated phages are tailed phages (Ackermann 2003). Infection by tailed phages is considered to be initiated via the attachment of the receptor-binding protein to bacterial surface molecules, so phage receptor-binding proteins are indispensable during phage infection (Vinga et al. 2006). However, the receptor-binding proteins of phages that infect Gram-positive bacteria have only been studied in a few phages (Vinga et al. 2006).

During the coevolutionary arms race between phages and bacteria, bacteria mainly change their surface structure to prevent phage attachment, whereas phages modify genes associated with phage host specificity and adsorption (Weitz et al. 2005; Uchiyama et al. 2011). Thus, phages tend to accumulate mutations in genes associated with adsorption at a faster rate than those in other parts of the genome, while bacteria accumulate mutations in genes related to surface structure modifications (Weitz et al. 2005; Yoichi et al. 2005; Paterson et al. 2010).

*S. aureus* phage S13′ was isolated from sewage and classified in the family *Podoviridae*, genus AHJD-like viruses (Takemura-Uchiyama et al. 2013). In the present study, another phage similar to phage S13′, phage S24-1, was isolated from local sewage. Phages S24-1 and S13′ may be involved in a host–parasite coevolutionary arms race. To the best of our knowledge, the genes that encode the receptor-binding proteins of *S. aureus* virulent phages have not been studied well. In the present study, therefore, we compared the genomes and the adsorption properties of phages S24-1 and S13′. Subsequently, we characterized the receptor-binding proteins of AHJD-like viruses using phage S24-1.

## Experimental Procedures

### Phages, bacterial strains, culture condition, culture media, and reagents

*S. aureus* phage S13′ was isolated from sewage influent water (Takemura-Uchiyama et al. 2013) and *S. aureus* phage S24-1 was isolated from the same sewage. *S. aureus* strain SA14 was used for phage amplification and for phage concentration measurements. The bacterial strains used in this study are described in Tables S1 and S2. The spot test used the *S. aureus* strains listed in Table S1, which have been described elsewhere (Takemura-Uchiyama et al. 2013). *Escherichia coli* was cultured in Lysogeny broth (LB) medium, whereas the other bacteria and phages were cultured in tryptic soy broth (TSB) at 37°C. The phage concentration was measured using the double-layer agar method (Takemura-Uchiyama et al. 2013). All of the culture media and reagents used in this study were obtained from Becton Dickinson and Company (Sparks, MD) and Nacalai Tesque (Kyoto, Japan), respectively, unless stated otherwise.

### Phage culture and purification

The phage was cultured with *S. aureus* strain SA14. After the complete lysis of *S. aureus*, the phage was purified by CsCl density-gradient ultracentrifugation, as described elsewhere (Takemura-Uchiyama et al. 2013). For the genomic DNA extraction and structural protein analysis, the phage band was diluted fourfold using AAS (0.1 mol/L ammonium acetate, 10 mmol/L NaCl, 1 mmol/L CaCl_2_, 1 mmol/L MgCl_2_, pH 7.2) and pelleted by ultracentrifugation (100,000*g*, 1 h, 4°C). For the electron and immunoelectron microscopy, the phage was dialyzed against AAS solution (1 h, 4°C).

### Electron microscopy

The samples were loaded onto formvar-carbon-coated copper grids and negatively stained with 2% uranyl acetate (pH 4.0). Electron micrographs were obtained using a Hitachi H-7100 transmission electron microscope (Hitachi, Ibaraki, Japan) at 100 kV.

### Characterization of phage S24-1 proteins

The phage pellet was suspended in 1 ×  Laemmli sodium dodecyl sulfate polyacrylamide gel electrophoresis (SDS-PAGE) sample buffer (2% SDS, 10% glycerol, 0.01% bromophenol blue, 0.1 mol/L dithiothreitol, 60 mmol/L Tris-HCl, pH 6.8) and boiled for 5 min. The phage structural proteins were electrophoresed using a SDS-PAGE gel. The proteins were stained using Coomassie brilliant blue (CBB), if required.

### Genome sequencing

The phage genome was extracted from the purified phage pellet, as described elsewhere (Uchiyama et al. 2011). The whole genome sequencing was conducted as described previously (Uchiyama et al. 2008). The sequence coverage redundancy was at least double. The potential open reading frames (*orf*s) were predicted using the gene predication tool GeneMark VIORIN (http://opal.biology.gatech.edu/GeneMark/) (Borodovsky et al. 2003) and were checked manually by considering the ribosome-binding site sequence. The genome sequences of phages S24-1 and S13′ were submitted to GenBank (accession numbers AB626963 and AB626962, respectively).

### Bioinformatic analysis

The genomic DNA sequences and the ORFs were analyzed using the BLAST program via the NCBI website (http://www.ncbi.nlm.nih.gov/blast/Blast.cgi) with an *E*-value threshold of 0.1 (Marchler-Bauer et al. 2011). The protein sequences were aligned using CLUSTALW and a phylogenetic tree was constructed with the neighbor-joining method, using the integrated software suite Molecular Evolutionary Genetics Analysis version 5 (MEGA5) (http://www.megasoftware.net/) (Tamura et al. 2011). Data were retrieved from GenBank (http://www.ncbi.nlm.nih.gov/genbank/).

### Spot test

The phage lytic activity was examined using a spot test (Kutter 2009), where 3 *μ*L of phage suspension (5.0 × 10^9^ pfu/mL) was spotted onto double-layered agar containing bacteria and incubated overnight at 37°C.

### Phage-adsorption assay with *S. aureus* cells

A 200 *μ*L aliquot of an appropriate overnight-cultured *S. aureus* strain was supplemented with 200 *μ*L of TSB and 100 *μ*L of phage (ca. 1.0 × 10^4^ pfu/mL). The mixture was then incubated for 3 min at 37°C. Next, after pelleting the *S. aureus* cells by centrifugation (20,000*g*, 30 sec), 400 *μ*L of the supernatant was mixed with 100 *μ*L of chloroform. The phage concentration that remained in the supernatant was measured using *S. aureus* strain SA14 and the phage-adsorption efficiency (percentage) was calculated, where that of the bacteria-untreated phage was 0%.

### Overexpression and purification of the recombinant protein

The coding sequence of the gene was amplified by polymerase chain reaction (PCR) with an appropriate primer set (Tables S3 and S4) using the phage genomic DNA as a template. The fragments were cloned into the expression vectors pCold II and pCold III (Takara Bio, Shiga, Japan). The protein was overexpressed in *E. coli* BL21, according to the manufacturer's instructions.

Bacterial cells were sonicated in a lysis solution (100 mmol/L sodium phosphate, 300 mmol/L NaCl, pH 7) and the cell lysate was incubated with Talon Metal Affinity Resin (Clontech Laboratories, Mountain View, CA) (90 min, 4°C). After washing with the lysis solution, the proteins were eluted with elution buffer (50 mmol/L sodium phosphate, 300 mmol/L NaCl, pH 6.0) and buffer supplemented with 5 and 350 mmol/L imidazole. After dialysis against phosphate-buffered saline (PBS; 137 mmol/L NaCl, 2.7 mmol/L KCl, 10 mmol/L Na_2_HPO_4_, 1.8 mmol/L KH_2_PO_4_, pH 7.2), the protein purity was examined by SDS-PAGE and the proteins were quantified using Bradford reagent (Sigma-Aldrich, St. Louis, MO).

### Anti-ORF16 antibody preparation

Female New Zealand white rabbits (11 weeks, 2 kg) (SLC Japan, Shizuoka, Japan) were immunized with rORF16s emulsified with Freund's adjuvant. This experiment was conducted with the approval of the Animal Experiment Committee of Kochi University (permit no. D-00022). After collecting the blood, rabbit serum was prepared and stored at −80°C until use.

### Western blotting

The proteins separated by SDS-PAGE were transferred to polyvinylidene difluoride (PVDF) membranes (Immobilon-P Membrane; Millipore, Billerica, MA) with blotting solution (10 mmol/L 3-[(3-Cholamidopropyl)dimethylammonio]-1-propanesulfonate (CHAPS) (pH 11), 10% methanol). To detect the 6× His-tag-fused protein, the membrane was incubated with anti-6× His-tag antibody (1:10,000; anti-His-tag horseradish peroxidase (HRP)-DirecT; Medical & Biological Laboratories, Nagoya, Japan). To detect ORF16, the membrane was incubated with anti-ORF16 rabbit serum (1:5000) and a secondary anti-rabbit immunoglobulin G, HRP-linked whole antibody (GE Healthcare, Little Chalfont, U.K.), as the primary and secondary antibodies, respectively. The immunoblot signals were detected using enhanced chemiluminescence (ECL) Western Blotting Detection Reagents (GE Healthcare) and visualized with X-ray films (Fuji Medical X-ray film, FUJIFILM Corporation, Tokyo, Japan).

### Immunoelectron microscopy

Anti-ORF16 rabbit serum was pretreated with *E. coli* BL21 acetone powder, which was prepared as described previously (Harlow and Lane 1988). The purified phage sample was incubated with anti-ORF16 antibody diluted in PBS (1:100) (30 min, room temperature). As a control, the purified phage sample was incubated with preimmunized rabbit serum (30 min, room temperature). The samples were loaded onto formvar-carbon-coated copper grids. The copper grid was washed with MilliQ water once and the grids were incubated with 12 nm Colloidal Gold-AffiniPure Goat Anti-Rabbit IgG (H+L) (Jackson ImmunoResearch Laboratories, West Grove, PA) in PBS (1:50) (30 min, 37°C). The grid was observed by electron microscopy.

### Mass spectrometric protein analysis by in-gel digestion

The proteins separated by SDS-PAGE were subjected to sample preparation for mass spectrometry, as described previously (Uchiyama et al. 2011). The prepared samples were analyzed using an AB SCIEX TOF/TOF 5800 System (AB Sciex, Foster City, CA). The protein database for phage S24-1 combined with *S. aureus* N315 (GenBank accession no. BA000018) was constructed locally for this experiment. Protein data for *S. aureus* were added to the database to increase the precision and accuracy of the protein identification. The data were analyzed using the Paragon method with ProteinPilot 3.0 (AB Sciex) based on the protein database (Shilov et al. 2007).

### Aggregation assay using ORF16-coated beads

An overnight bacterial culture diluted in TSB (50 *μ*L) was loaded into the wells of a sterile flat-bottomed polystyrene 96-well plate (F96 MicroWell Plates; Thermo Fisher Scientific, Roskilde, Denmark) and the optical density was adjusted to ca. 0.1 at 595 nm using a Multiskan JX spectrophotometer (Thermo Labsystems, Stockholm, Sweden).

Next, 150 *μ*g of the protein (either BSA or rORF16) was conjugated to 20 mg of silica beads in 1 ml volume using an Affinity Beads Kit (Sumitomo Barkelite, Tokyo, Japan), according to the manufacturer's protocol. The beads were also prepared without protein, serving as a control. The beads were lyophilized by a freeze-drier (Neocool Unit; Yamato Scientific, Tokyo, Japan), and stored at 4°C until use. The freeze-dried beads were rehydrated with 700 *μ*L of PBS before use. A volume of 50 *μ*L of the bacterial suspension was added to 25 *μ*L of the beads in the wells of a sterile U-bottomed polystyrene 96-well plate (U96 MicroWell Plates; Thermo Fisher Scientific), which was incubated with shaking for 1 min at room temperature. The aggregation was visually examined.

### Phage neutralization assay using anti-ORF16 rabbit antibody

In this assay, 0.2 mL of phage (ca. 1.0 × 10^4^ pfu/mL) was mixed with 0.2 mL of the anti-ORF16 rabbit serum, which was serially diluted with TSB. As controls, 0.2 mL of the preimmunized rabbit serum or TSB was also added to 0.2 mL of phage suspension. The mixture was incubated (30 min, 37°C) with shaking and the phage concentration was measured. The concentration of the phage alone was set to 100%.

### Treatment of *S. aureus* cells with heat and various chemicals

After washing *S. aureus* strain SA14 using PBS three times, the *S. aureus* pellet was subjected to different treatments as follows: heat (15 min, 121°C), 4% SDS (30 min, 100°C), 90% trichloroacetic acid (TCA) (30 min, room temperature), 100 *μ*g/mL of proteinase K (30 min, 50°C), phenol–chloroform (1:1) (30 min, room temperature), n-butanol (30 min, room temperature), 1% Triton X-100 (50°C, 30 min), 0.1 mol/L NaOH (2 days, room temperature), and 49% hydrofluoric acid (HF) (2 days, 4°C). After the treatments, the pellets were washed five times with PBS. As a control, *S. aureus* strain SA14 was washed with PBS three times.

### Preparation of teichoic acids

The *S. aureus* lipoteichoic acids (LTAs) were obtained from Sigma-Aldrich. The wall teichoic acids (WTAs) of *S. aureus* strain SA14 were extracted using a NaOH treatment procedure, as described elsewhere (Xia et al. 2010). After extraction using the NaOH treatment, the sample was neutralized with HCl at pH 7.0. The concentrated WTA suspension was dialyzed against water and lyophilized by the freeze-drier.

### Extraction of peptidoglycans

The peptidoglycans of *S. aureus* strain SA14 were prepared as described previously (Gründling et al. 2006).

### Identification of the bacterial component binding to the phage

The receptor molecules binding to the phage were examined using the method described in “Phage-adsorption assay with *S. aureus* cells.” First, the phage adsorption was measured using *S. aureus* treated with heat and various chemicals, as described in “Treatment of *S. aureus* cells with heat and various chemicals.” Approximately 2.0 mg of *S. aureus* was suspended in 500 *μ*L of PBS and used in the experiments. Second, the phage adsorption was measured in the presence of WTAs, rORF16, and BSA. The WTAs and the proteins were suspended in PBS and used in the experiment. The phage adsorption was measured after supplementation with WTAs, rORF16, and BSA.

### Analysis of the *S. aureus* cell wall molecule binding to ORF16

The ORF16-binding molecule derived from *S. aureus* was examined. First, an aggregation assay using rORF16-bound beads was conducted with *S. aureus* and with the *S. aureus* samples treated with heat and various chemicals, as described above. The aggregation assay using rORF16-bound beads was also conducted with 10 *μ*g/*μ*L of the peptidoglycans, LTAs, or WTAs suspended in PBS.

The ORF16-binding molecules derived from *S. aureus* were also examined by western blotting, where ca. 2.0 mg of the *S. aureus* samples treated with heat and various chemicals were mixed with 20 *μ*L of PBS or rORF16 (50 *μ*g/mL). The mixture was incubated with shaking at 37°C for 5 min. The bacteria were pelleted by centrifugation (20,000*g*, 1 min) and the supernatant and the pellet were collected separately. The bacterial pellet was then washed with PBS twice and suspended in PBS. An equal volume of 2× Laemmli SDS-PAGE sample buffer was added to the supernatant or the bacterial suspension. The samples were heated (100°C, 5 min) and subjected to western blotting using the anti-6× His-tag antibody described above.

### Examination of the binding activities of rORF16 and truncated rORF16s by western blotting

The binding of rORF16 or terminally deleted rORF16s with *S. aureus* strain SA14 were examined by western blotting, as described in the second part of “Analysis of the *S. aureus* cell wall molecule binding to ORF16.” The samples were subjected to western blotting using the anti-6× His-tag antibody, as described above.

### Statistical analysis

The sextuplicated data were collected, and were analyzed using a Mann–Whitney *U* test with GraphPad InStat (version 3; GraphPad Software Inc., La Jolla, CA).

## Results

### Comparative genomic analysis of phages S24-1 and S13′

**Figure 1:**
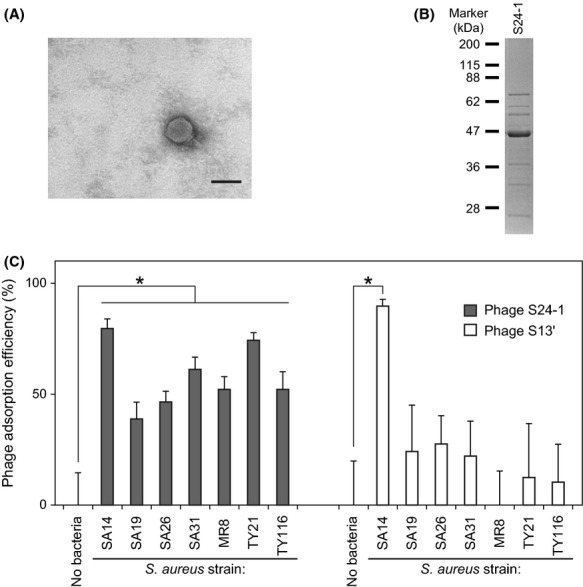
Characteristics of phage S24-1. (A) Transmission electron microscopy. The bar represents 50 nm. (B) Structural protein analysis. The phage structural proteins were separated electrophoretically by SDS-PAGE (12.5% gel), together with molecular weight markers (prestained Protein Markers [Broad Range] for SDS-PAGE; Nacalai Tesque). The virion protein band pattern of phage S24-1 was similar to that of phage S13′ (Takemura-Uchiyama et al. 2013). (C) Adsorption efficiencies. The *Staphylococcus aureus* strains used in this assay were insensitive to the lysis-from-without activities of phage S13′ (Table S1). Phage adsorption was examined using these *S. aureus* strains. *S. aureus* strain SA14, which both phages S24-1 and S13′ could adsorb efficiently, was used as a positive control. Phages alone, which are indicated as “No bacteria,” were used as a negative control. The mean values and standard deviations are shown as a bar graph with error bars (*n* = 6). Statistically significant differences are indicated by an asterisk (*P *<* *0.01).

Phage S24-1 was classified in the family *Podoviridae*, genus AHJD-like viruses, because of its similarity in morphology and virion proteins to those of phage S13′ (Fig. 1A and B) (King et al. 2012; Takemura-Uchiyama et al. 2013). Sequencing of the genomes showed that phage S24-1 (18,168 bp) had a slightly smaller genome than phage S13′ (18,186 bp). Both phages S24-1 and S13′ were predicted to have 21 *orf*s (Tables S5 and S6). In phages S24-1 and S13′, ORF19 was identified as a major capsid protein, according to the results of the N-terminal protein sequencing in our previous study (Fig. 1B) (Takemura-Uchiyama et al. 2013). The protein BLAST and domain analyses suggested that ORFs 10, 11, 12, and 15 were DNA polymerase, tail lytic protein, holin, and endolysin, respectively, in both phages S24-1 and S13′.

Nucleotide BLAST analysis of phages S24-1 and S13′ showed that they had ca. 96% shared identity. The sequence dissimilarity occurred at around 12.5–13.5 kbp in the genomes of phages S24-1 and S13′, where *orf16* was located. Moreover, ORF16 of phages S24-1 and S13′, and homologous ORFs, were conserved among the AHJD-like viruses, although their function remains unknown. According to the phylogenetic analysis of the homologous ORFs in AHJD-like viruses, ORF16 of phage S24-1 was highly diverged from AHJD-like viruses, whereas the other ORFs appeared to be relatively similar (Fig. S1). The coevolutionary arms race between phages and bacteria leads to the accumulation of many mutations, particularly in genes that encode proteins related to adsorption and host specificity (Paterson et al. 2010). Our results suggested that ORF16 was likely to be associated with adsorption and host specificity.

### Adsorption differences between phages S24-1 and S13′

During infection, the tailed phage attaches to the bacterial surface and makes a hole via a tail lytic protein before the injection of genomic DNA. If a large number of phages attach to the bacterial surface, bacteriolysis occurs independently of phage multiplication because of the combined lytic effects of the tail lytic proteins. This form of bacteriolysis is known as “lysis-from-without” (Abedon 2011). The activity level of lysis-from-without can be measured based on spot formation by spotting phages onto a double-layered agar plate containing bacteria. Spot formation does not occur without attachment of phages to the bacterial cells, even if there are high numbers. The putative tail lytic proteins (ORF11s) were identical in phages S24-1 and S13′, so their spot-forming spectra are likely to reflect their phage-adsorption capacities.

Analysis of the lysis-from-without effects of phages S24-1 and S13′ using a spot test with 89 clinically isolated strains showed that phages S24-1 and S13′ produced 100% (89/89) and 93.3% (83/89) lytic spectra, respectively (Table S1). Phage S13′ had no lytic activity against six *S. aureus* strains, i.e., SA19, SA26, SA31, MR8, TY21, and TY116. Subsequently, the adsorption efficiencies of phages S24-1 and S13′ were examined using the phage S13′-insensitive *S. aureus* strains (Fig. 1C), which showed that phage S24-1 was adsorbed more efficiently by these *S. aureus* strains than phage S13′. Phage S13′ was only adsorbed efficiently by *S. aureus* strain SA14. This result, as well as the distinct divergence of phage S24-1 ORF16 compared with other AHJD-like viruses, suggested that phage S24-1 was better adapted to this set of various *S. aureus* strains than phage S13′. Unfortunately, information is not available on the receptor-binding protein in other AHJD-like viruses. Thus, we investigated the putative receptor-binding protein, using phage S24-1 and its ORF16.

### ORF16, a structural component in phage S24-1

**Figure 2:**
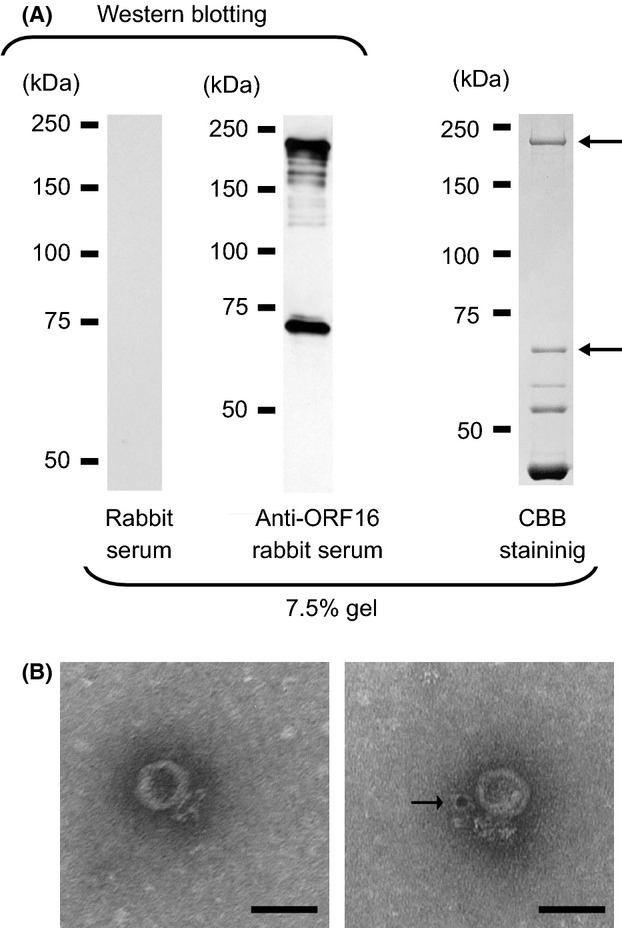
(A) Analysis of the structural proteins of phage S24-1. Western blot analysis of ORF16 against the structural proteins of phage S24-1 (left and middle). The proteins separated by SDS-PAGE were visualized by CBB staining (right). The protein bands indicated by arrows were identified as ORF16 by mass spectrometry (see Fig. S2). (B) Immunoelectron microscopic analysis of ORF16 against phage S24-1. A control electron micrograph is shown on the left. An electron micrograph of phage S24-1 treated with the anti-ORF16 rabbit antibody is shown on the right, where a gold particle appears to be attached to the vicinity of the phage tail, which is indicated by an arrow. The bars represent 50 nm.

The presence of ORF16 in phage S24-1 structural proteins was examined by western blotting using anti-ORF16 rabbit antibody (Fig. 2A). Protein bands were detected at ca. 70 kDa and ca. 210 kDa, which also corresponded to the highest and second-highest protein bands in the CBB R-250-stained SDS-PAGE gels. Moreover, these two proteins were digested by trypsin and were analyzed by mass spectrometry. The mass spectra of the peptides derived from the two protein bands were almost identical, which suggested that both were ORF16 (Figs. 2A and S2). These results indicated that ORF16 was a structural component of phage S24-1, which was SDS-resistant and trimerized. The location of ORF16 in the phage structure was analyzed by immunoelectron microscopy using the anti-ORF16 rabbit antibody (Fig. 2B). Treatment of the phage with anti-ORF16 rabbit antibody showed that gold particles bound to the structural protein adjacent to the phage tail, whereas the phage treated with preimmunized rabbit antibody had no gold particles on it. Thus, ORF16 was a structural protein, possibly a tail component.

### ORF16 binding activity

**Figure 3:**
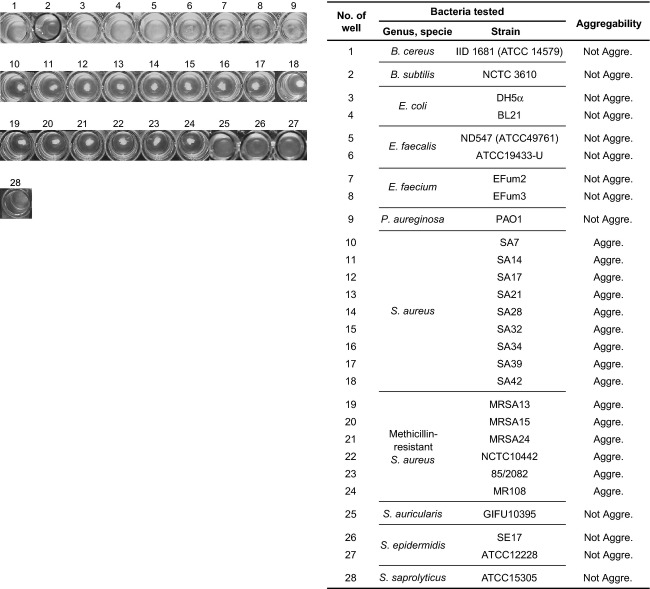
Aggregation assay against various bacteria using rORF16-bound beads. The rORF16-bound beads were incubated with bacteria and aggregation or nonaggregation was assessed visually. Wells where the rORF16-bound beads reacted with various bacteria are shown in the left column. The numbers from 1 to 28 are shown on top of the wells. The number of the well corresponds to the table on the right-hand side. Aggregation and nonaggregation are abbreviated as “Aggre.” and “Not Aggre.,” respectively.

The majority of antibodies can adsorb to protein A, which surrounds *S. aureus*, so an assay system that lacked antibody was desirable for analyzing the binding activity of ORF16. Thus, we established a novel aggregation assay system based on recombinant ORF16 of phage S24-1 (rORF16)-bound beads (Fig. S3). Using this aggregation assay system, we examined the binding capacity of ORF16 to various bacteria, including methicillin-sensitive *S. aureus*, MRSA, non-*aureus* staphylococci, and other Gram-positive and-negative bacteria (Fig. 3). The rORF16-bound beads only produced aggregates with *S. aureus*, whereas no aggregates were produced with the other bacteria. Phage S24-1 also formed no plaques and had no lysis-from-without activity against non-*aureus* staphylococci and the Gram-positive and-negative bacteria tested in this study (Table S2). Thus, the binding specificity of ORF16 appeared to correspond to the adsorption specificity of phage S24-1. Therefore, ORF16 was considered to have a specific binding activity with *S. aureus*, which may be important for the host specificity of phage S24-1.

### Importance of ORF16 in phage S24-1 infection

**Figure 4:**
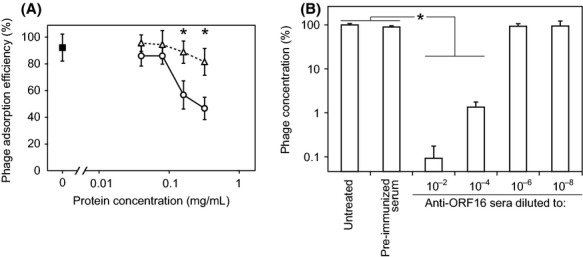
Role of ORF16 in phage S24-1 adsorption. The means and standard deviations are shown in the graphs (*n* = 6). The asterisks in the graphs indicate statistically significant differences (*P *<* *0.01). (A) Phage-adsorption interference assay using rORF16. The white triangles and circles indicate the phage-adsorption efficiencies in the presence of BSA and ORF16, respectively. The black box indicates the phage-adsorption efficiency without any protein supplements (control). The phage-adsorption efficiencies were compared for the BSA and rORF16 treatments at the same protein concentrations. (B) Phage-adsorption interference assay using anti-ORF16 rabbit serum. The phage concentration was measured after incubation with diluted anti-ORF16 rabbit sera or preimmunized rabbit serum (30 min, 37°C). The untreated phage concentration was set at 100%.

ORF16 had a binding affinity for *S. aureus*, so we tested whether ORF16 was essential during phage adsorption. First, the adsorption efficiency of phage S24-1 was measured in the presence of rORF16s. The rORF16s appeared to interfere with phage adsorption in a concentration-dependent manner, whereas phage adsorption was not affected by bovine serum albumin (BSA) (Fig. 4A). The phage titers were also measured after treating the phages with anti-ORF16 rabbit sera. Treatment with a higher concentration of anti-ORF16 rabbit sera produced a lower phage titer (Fig. 4B). Therefore, ORF16 appeared to be essential for phage adsorption.

### Binding of *S. aureus* components to ORF16

We investigated the ORF16 receptor molecule on the cell walls of *S. aureus*. The cell wall of *S. aureus* contains proteins, peptidoglycans, LTAs, and WTAs (Swoboda et al. 2010). Various treatments with heat or chemicals were used to reduce the components of *S. aureus* to protein and teichoic acids. To prepare protein-, LTA-, and WTA-depleted cells, *S. aureus* cells were treated with: heat, SDS, protease K or Triton X-100; butanol or phenol–chloroform; and NaOH or HF, respectively (Morath et al. 2001; Sadovskaya et al. 2005; Xia et al. 2010).

**Figure 5:**
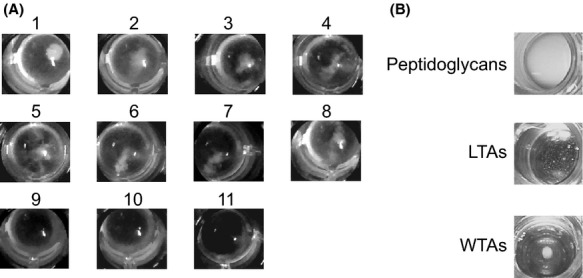
Investigation of the ORF16-binding molecule. (A) Aggregation assay against protein-and teichoic acid-depleted *Staphylococcus aureus* using rORF16-bound beads. The results of the aggregation assay with the following *S. aureus* samples are shown: untreated *S. aureus* cells in well 1, *S. aureus* autoclaved cells in well 2, *S. aureus* cells treated with SDS in well 3, *S. aureus* cells treated with trichloroacetic acid (TCA) in well 4, *S. aureus* cells treated with proteinase K in well 5, *S. aureus* cells treated with phenol–chloroform in well 6, *S. aureus* cells treated with n-butanol in well 7, *S. aureus* cells treated with Triton X-100 in well 8, *S. aureus* cells treated with NaOH in well 9, and *S. aureus* cells treated with HF in well 10. In well 11, the rORF16-bound beads were mixed with PBS only. Wells 1–8 contains aggregates, whereas wells 9–11 do not contain aggregates. (B) Aggregation assay against cell wall molecules using rORF16-bound beads. The rORF16-bound beads were mixed with *S. aureus* peptidoglycans, LTAs, and WTAs. An aggregate was observed in the mixture with WTAs, whereas aggregates were not observed in the mixtures with peptidoglycans and LTAs.

The binding ability of rORF16 was then examined in the treated *S. aureus* samples using the aggregation assay with rORF16-bound beads (Fig. 5A). The aggregation assays showed that the rORF16-bound beads did not aggregate with WTA-depleted (i.e., NaOH-and HF-treated) *S. aureus*, whereas they aggregated with the LTA-and protein-depleted (i.e., treated with heat or other chemicals) *S. aureus* samples. In addition, the rORF16 binding activity was also examined using another experimental method. Mixing of the rORF16 and protein-, WTA-, or LTA-depleted *S. aureus*, and the unbound rORF16 was examined by the western blotting. The antibody-binding activity of protein A does not influence the results in this experimental setting. The results of the western blot analyses of the rORF16-binding activity were similar to those obtained using the aggregation assays with rORF16-bound beads (Fig. S4).

The aggregation assay using the rORF16-bound beads was also conducted with peptidoglycans, LTAs, and WTAs (Fig. 5B). The WTAs produced aggregation, whereas the others did not. Thus, the ORF16 receptor molecule was considered to be WTAs.

### *S. aureus* receptor to phage S24-1

**Figure 6:**
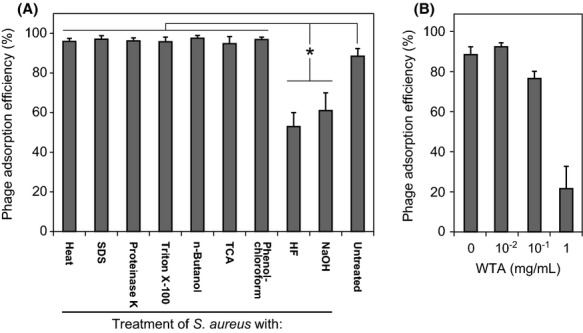
*Staphylococcus aureus* cell wall molecule to phage S24-1 adsorption. The means and standard deviations are shown in the graphs (*n* = 6). The asterisks in the graphs indicate statistically significant differences (*P *<* *0.01). (A) Phage-adsorption efficiencies against *S. aureus* treated with heat and various chemicals. The phage-adsorption efficiencies were measured against *S. aureus* samples treated with heat and various chemicals. (B) Phage-adsorption efficiency in the presence of WTAs. The phage-adsorption efficiency reduced as the concentration of WTAs increased.

The *S. aureus* surface receptor molecules for S24-1 were examined. First, the phage-adsorption efficiency was examined in heat-and chemically treated *S. aureus* cells (Fig. 6A). The adsorption efficiencies of phage S24-1 with WTA-depleted (i.e., NaOH-and HF-treated) *S. aureus* were significantly lower than those with *S. aureus* after the other treatments. In addition, the disruption of proteins in *S. aureus* cells appeared to increase phage adsorption. Moreover, the adsorption efficiency of phage S24-1 was examined in the presence of WTAs (Fig. 6B), which interfered with phage adsorption in a concentration-dependent manner. Thus, the phage S24-1 receptor molecule was considered to be a WTA(s).

### Analysis of the ORF16 protein sequence essential for the binding activity of phage S24-1

We analyzed ORF16 of phage S24-1 and S13′ using Conserved Domain Search via the National Center for Biotechnology Information (NCBI) website, which showed that the C-terminal regions contained the PHA01818 protein domain with E-values of 1.8e-4 and 1.7e-4, respectively. The PHA01818 domain is also considered to be conserved in the hypothetical proteins of staphylococcal lytic phages that belong to the genus of Twort-like viruses, including ORF68 of *Staphylococcus* phage K, ORF017 of *Staphylococcus* phage Twort, and ORF017 of *Staphylococcus* phage G1. The conserved PHA01818 domain was also found in the ORF16 homologs of AHJD-like viruses. The function of the PHA01818 domain remains unknown.

**Figure 7:**
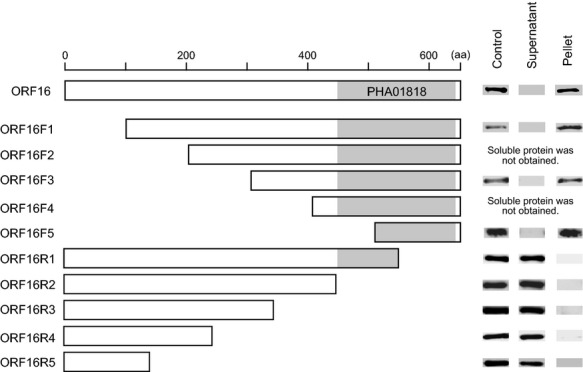
Examination of the essential ORF16 protein sequence required for its binding activity. Various terminally deleted rORF16s were prepared, although some proteins were not obtained in a soluble form. Their binding activities were examined. After incubating *Staphylococcus aureus* with the terminally deleted rORF16, the supernatant and *S. aureus* cells were separated by centrifugation. The supernatant and *S. aureus* were subjected to western blotting. The ORF16 itself was used as a control. Diagrams of the N-terminally or C-terminally deleted ORF16s are shown in the left column. The PHA018018 protein domain is highlighted in gray. The western blotting results are shown in the column on the right.

The ORF16 protein sequence essential for the binding activity was analyzed using terminally deleted ORF16s. After treatment of the soluble terminally deleted ORF16s with *S. aureus*, western blotting was conducted against *S. aureus* cells and the supernatant (Fig. 7). All of the C-terminally deleted ORF16s lacked binding activity to *S. aureus*, whereas the N-terminally deleted ORF16s retained the binding activity. ORF16F5, which included ca. 140 amino acids from the C-terminal end with a slight loss of the integrity of the PHA01818 domain, still had a binding activity with *S. aureus*. Thus, the C-terminal region of ORF16 was considered to be essential for its binding activity.

## Discussion

The coevolutionary arms race between bacteria and phages facilitates their extremely rapid evolution and turnover (Comeau and Krisch 2005). Interestingly, the AHJD-like viruses can be isolated throughout the world (Vybiral et al. 2003; Kwan et al. 2005; Son et al. 2010) but they are genetically very similar. This suggests that these *S. aureus* phages may be good model phages for studying the coevolutionary arms race between *S. aureus* and phages on a global scale. Given the genetic differences in the biological activities of phages S24-1 and S13′ and the genetic divergence of phage S24-1, it is thought that phage S24-1 was engaged in an evolutionary process at a higher rate than phage S13′ in the past.

The short-tailed phages, including phages P22, HK620, Det7, φ29, K1F, and SF370.1, possess tail spike proteins with *β*-helical and trimeric structures, which function as receptor-binding proteins (Steinbacher et al. 1994; Stummeyer et al. 2005; Walter et al. 2008; Xiang et al. 2009; Casjens and Molineux 2012). Many of these proteins form homotrimers and are SDS-resistant (Mitraki et al. 2002). In phage S24-1, ORF16 was considered to form homotrimers with SDS resistance. The ORF16 of phage S24-1 may form a spike-like structure, similar to that of other short-tailed phages (Casjens and Molineux 2012).

The teichoic acids function as phage receptors for other families of phages such as siphophages and myophages (Kaneko et al. 2009; Xia et al. 2011). In the present study, we showed that *S. aureus* WTA was the receptor molecule for ORF16 of podophage S24-1. The specific binding of ORF16 to *S. aureus* WTA (20–60% of the total cell wall mass; Hancock and Baddiley 1985) restricted the host range of phage S24-1 to *S. aureus*. Moreover, the proteins in *S. aureus* cells seemed to interfere with the phage adsorption. The proteins in *S. aureus* may disturb the charge or structure of *S. aureus* WTAs, thereby decreasing the affinity of ORF16 for *S. aureus* WTAs. Thus, the cell wall proteins of *S. aureus* may protect against phage adsorption.

The PHA01818 domain is common in the proteins of Twort-like viruses, as well as being present in ORF16 of phage S24-1 and in some ORF16 homologs in other AHJD-like viruses, whereas it is not present in the ORFs of siphoviruses (e.g., phage φSLT). According to our functional study of ORF16 using terminally deleted ORF16s, the PHA01818 domain is probably associated with the *S. aureus* adsorption activity. We also tested the adsorption activity of other proteins that contain the PHA01818 domain. *Staphylococcus* phage K, a member of the Twort-like viruses, contains an ORF with a PHA01818 domain (i.e., ORF68 of staphylococcal phage K, K_ORF68). A recombinant K_ORF68 protein was expressed in *E. coli* and the binding activity was examined (Fig. S5). Unfortunately, K_ORF68 had no binding activity with *S. aureus*, regardless of the presence of cationic ions. Thus, K_ORF68 may require partner molecules to function properly, or may not be associated directly with adsorption. Based on this evidence, the protein primary structure did not seem to be essential in the protein function, particularly phage adsorption. The ORF16s and related proteins suggest other conserved protein domains in the primary structure.

Phages and phage-derived molecules have been used widely in nanotechnology, therapy, and the life sciences (Tarascon 2009; Lu and Koeris 2011; Singh et al. 2011, 2012; Hyman 2012). Phage receptor-binding proteins have been used as biosensors and to eliminate bacteria colonization for prophylactic purposes (Waseh et al. 2010). In the present study, the aggregation assay (in Figs. 3 and S3) suggests a possible use of phage receptor-binding proteins in a bacterial detection system.

In this study, a genomic comparative and phage infection study of phages S13′ and S24-1 suggested that ORF16 is associated with adsorption and host specificity. Analysis of ORF16 of phage S24-1, ORF16, which is likely to be present near the tail, shows that it functions as an adsorption molecule in phage S24-1. Considering these findings, ORF16 could be an essential receptor-binding protein in phage S24-1. In future, we hope that the phage receptor-binding protein will be investigated further and that it will facilitate further progress in the study of phage–host interactions, and the application of phages and phage-derived products.

## Supporting Information

**Figure S1**Phylogenetic analysis of phage S24-1 ORFs in AHJD-like viruses. Phylogenetic trees of major capsid proteins (ORF19) (left), tail lytic protein (ORF11) (middle), and receptor-binding protein (ORF16) (right). The homologous ORFs in AHJD-like viruses were used in the analysis. First, the major capsid protein is typically used for phage phylogenetic analysis. Phages S24-1 and S13′ were considered to be more related to each other compared with the other AHJD-like viruses. Thus, phages S24-1 and S13′ may have evolved locally from the same origin. Next, the genetic distances of the tail lytic proteins and the major capsid proteins appeared to be smaller among the other AHJD-like viruses. By contrast, the putative receptor-binding protein (ORF16) of phage S24-1 was highly diverged from those of the other AHJD-like viruses, including phage S13′. The ORF data were obtained from the genome data: phage φ44AHJD (accession number AF513032), phage SAP-2 (accession number EU136189), Bacteriophage 66 (accession number AY954 949), and phage φP68 (accession number AF513033). **Figure S2.**Mass spectrometric analysis of two proteins from phage S24-1. (A) The structural proteins of phage S24-1. The structural proteins of phage S24-1 were separated using SDS-PAGE (7.5% gel) and visualized by CBB staining (see Fig. 2). The protein bands boxed in gray were digested by trypsin and analyzed by mass spectrometry. (B) Mass spectrometric analysis of the digested upper protein band. (C) Mass spectrometric analysis of the digested lower protein band. The mass spectra of the digested protein are shown on the left. The tables on the right show the mass spectrometry results. The peak numbers in the mass spectra correspond to those listed in the table. “Precursor molecular weight” is abbreviated as “Prec. MW.” **Figure S3.**Aggregation assay using rORF16-bound beads. (A) Schematic diagram of the aggregation assay system. If the protein had an affinity for the bacteria, aggregation with the bacteria was observed in the mixture (left). If the protein had no affinity for the bacteria, aggregation was not observed in the mixture (right). (B) Establishment of an aggregation assay system using rORF16-bound beads. rORF16-bound beads, BSA-bound beads, and beads only were prepared. Overnight cultures of *S. aureus* SA14 or *Enterococcus faecalis* EF24 were prepared. Twofold serial dilutions of the beads and bacteria were prepared using PBS. The beads were mixed with the bacteria and the aggregability was examined. Aggregation was only observed in the mixture of rORF16-bound beads with S. *aureus,* in a concentration-dependent manner. **Figure S4.**Assessment of the rORF16 binding affinity with *S. aureus* samples treated with heat and various chemicals. After rORF16 was mixed with *S. aureus* samples treated with heat and various chemicals, the supernatants and bacterial cell pellets were separated by centrifugation. The samples were subjected to rORF16 detection by western blotting. The treatment group number is shown at the top for each result. The types of treatments and their designated numbers are as follows: untreated in No. 1, autoclaved in No. 2, SDS in No. 3, trichloroacetic acid (TCA) in No. 4, phenol– chloroform in No. 5, n-butanol in No. 6, Triton X-100 in No. 7, NaOH in No. 8, and HF in No. 9 “has been replaced with” proteinase K in No. 5, phenol– chloroform in No. 6, n-butanol in No. 7, Triton X-100 in No. 8, NaOH in No. 9, and HF in No. 10. The result with ORF16 only is shown at the top left. Overall, the results agree with the aggregation assay results shown in Figure 5. Unfortunately, *S. aureus* cells treated with proteinase K did not produce any bands from the pellet or the supernatant (data not shown), probably because proteinase K residuals appeared to digest the treated ORF16, even after thorough washing of the bacterial cells. The NaOH and HF-treated samples (Nos. 8 and 9) had no binding affinity with *S. aureus.* **Figure S5.**Assessment of the binding activity using a recombinant ORF68 of phage K. The binding activity of phage K ORF68 was examined by western blotting using the recombinant proteins KORF68c-his (ORF68 with a 6 His-tag at the C-terminal) and KORF68n-his (ORF68 with a 6 His-tag at the N-terminal). *S. aureus* SA14 was sensitive to phage K infection. After incubation of the recombinant K_ORF68s with *S. aureus* strain SA14, the presence of the recombinant ORF68s in the solution or *S. aureus* cells was examined by western blotting using the anti-6 His antibody. KORF68c-his and KORF68n-his had no binding activity with *S. aureus*, regardless of the presence of cationic ions such as MgCl2 and CaCl2.

Table S1 The phage lysis activities of phages S13′ and S24-1 against 89 *S. aureus* strains.

Table S2 Bacterial strains used in this study and their phage S24-1 infectivity.

Table S3 List of primers used in this study.

Table S4 PCR products and primer lists.

Table S5 Annotation of the phage S13′ genome.

Table S6 Annotation of the phage S24-1 genome.
