# Supplementary material for: In silico analysis of AHJD-like viruses, Staphylococcus aureus phages S24-1 and S13′, and study of phage S24-1 adsorption
Source: Microbiologyopen. 2014 Mar 4;3(2):257–70. doi: 10.1002/mbo3.166 (PMC3996573; doi:10.1002/mbo3.166)
Supplement: Supplementary file 1 — Figure S1Phylogenetic analysis of phage S24-1 ORFs in AHJD-like viruses. Phylogenetic trees of major capsid proteins (ORF19) (left), tail lytic protein (ORF11) (middle), and receptor-binding protein (ORF16) (right). The homologous ORFs in AHJD-like viruses were used in the analysis. First, the major capsid protein is typically used for phage phylogenetic analysis. Phages S24-1 and S13′ were considered to be more related to each other compared with the other AHJD-like viruses. Thus, phages S24-1 and S13′ may have evolved locally from the same origin. Next, the genetic distances of the tail lytic proteins and the major capsid proteins appeared to be smaller among the other AHJD-like viruses. By contrast, the putative receptor-binding protein (ORF16) of phage S24-1 was highly diverged from those of the other AHJD-like viruses, including phage S13′. The ORF data were obtained from the genome data: phage φ44AHJD (accession number AF513032), phage SAP-2 (accession number EU136189), Bacteriophage 66 (accession number AY954 949), and phage φP68 (accession number AF513033). Figure S2.Mass spectrometric analysis of two proteins from phage S24-1. (A) The structural proteins of phage S24-1. The structural proteins of phage S24-1 were separated using SDS-PAGE (7.5% gel) and visualized by CBB staining (see Fig. 2). The protein bands boxed in gray were digested by trypsin and analyzed by mass spectrometry. (B) Mass spectrometric analysis of the digested upper protein band. (C) Mass spectrometric analysis of the digested lower protein band. The mass spectra of the digested protein are shown on the left. The tables on the right show the mass spectrometry results. The peak numbers in the mass spectra correspond to those listed in the table. “Precursor molecular weight” is abbreviated as “Prec. MW.” Figure S3.Aggregation assay using rORF16-bound beads. (A) Schematic diagram of the aggregation assay system. If the protein had an affinity for the bacteria, aggregation with the bacteria [file mbo30003-0257-sd1.pdf]

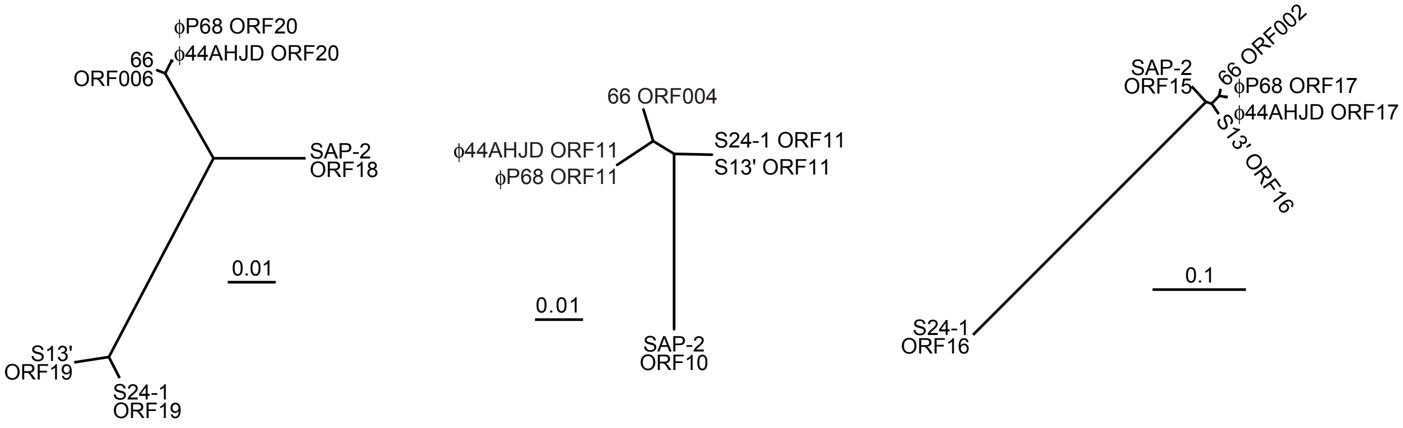

Fig. S1. Phylogenetic analysis of phage S24-1 ORFs in AHJD-like viruses. Phylogenetic trees of major capsid proteins (ORF19) (left), tail lytic protein (ORF11) (middle), and receptor-binding protein (ORF16) (right). The homologous ORFs in AHJD-like viruses were used in the analysis. First, the major capsid protein is typically used for phage phylogenetic analysis. Phages S24-1 and S13′ were considered to be more related to each other compared with the other AHJD-like viruses. Thus, phages S24-1 and S13′ may have evolved locally from the same origin. Next, the genetic distances of the tail lytic proteins and the major capsid proteins appeared to be smaller among the other AHJD-like viruses. By contrast, the putative receptor-binding protein (ORF16) of phage S24-1 was highly diverged from those of the other AHJD-like viruses including phage S13′. The ORF data were obtained from the genome data: phage ϕ44AHJD (accession number AF513032), phage SAP-2 (accession number EU136189), Bacteriophage 66 (accession number AY954949), and phage ϕP68 (accession number AF513033).

A

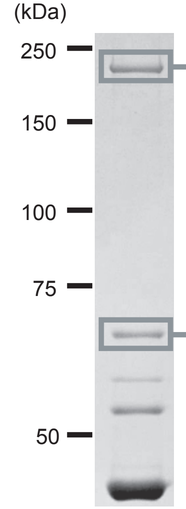

B

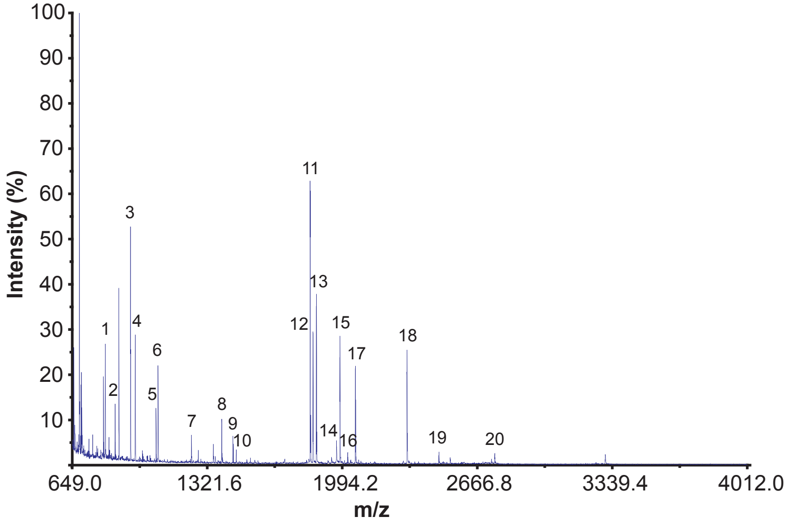

Peak	Confidence	Identified ORF	Matched peptide sequence	deltaMass	Prec. MW.
1	99	ORF16	LIEVLAR	0.0365	812.5485
2	84	ORF16	LVQIAYR	0.0328	861.54
3	99	ORF16	FVTGFGGVR	0.0386	938.536
4	99	ORF16	YGGGLIQVR	0.0264	961.5609
5	2	ORF16	SYYDYISR	0.0335	1065.5096
6	96	ORF16	YTPFYDDR	0.0242	1075.4857
7	99	ORF16	NAVNQSILIDR	0.0201	1241.6926
8	< 1	ORF16	TQQLGGYQELTR	0.0166	1392.7167
9	99	ORF16	EPEGIYLYVNPR	0.0104	1448.7407
10	8	ORF16	IVEGGHGTNIALER	0.0112	1464.7797
11	99	ORF16	YFDDIRPFLDEIYK	0.0185	1832.9166
12	99	ORF16	QNLLYGFFQTGEYER	0.0201	1846.8727
13	99	ORF16	QNLLYGFFQTGEYER	0.0082	1863.8877
14	5	ORF16	DNFREPEGIYLYVNPR	0.2396	1964.2186
15	99	ORF16	DNFREPEGIYLYVNPR	0.003	1980.9727
16	< 1	ORF16	RQNLLYGFFQTGEYER	-0.0333	2019.9467
17	99	ORF16	GIGDAGAWLQVSSGNVSGEVR	-0.001	2058.0117
18	99	ORF16	VYDYYLNDVSFEHGVENPR	-0.0079	2315.0417
19	96	ORF16	VMDDFPYINNDAGWFLFVSPK	-0.0273	2474.1348
20	56	ORF16	ENTPDRPMQGIAVYGDDLYWLSGR	-0.0503	2752.2417

C

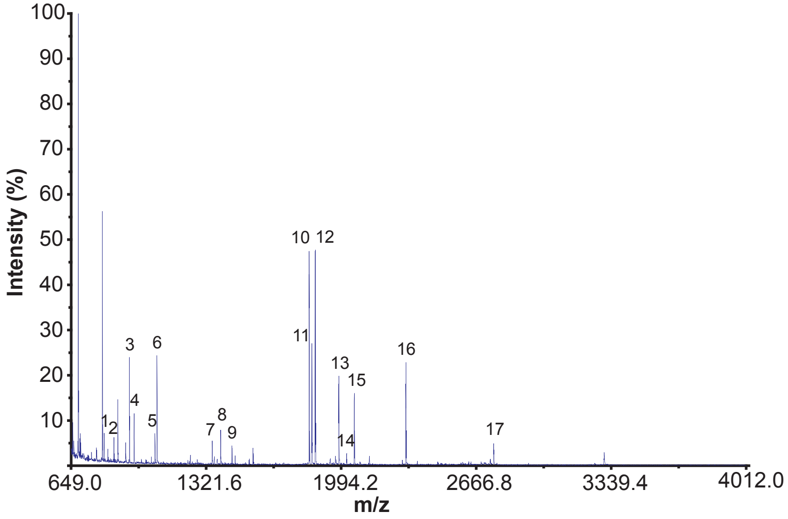

Peaks	Confidence	Identified ORF	Matched peptide sequence	deltaMass	Prec. MW.
1	99	ORF16	LIEVLAR	0.0164	812.5284
2	73	ORF16	LVQIAYR	0.016	861.5233
3	99	ORF16	FVTGFGGVR	0.0399	938.5374
4	99	ORF16	YGGGLIQVR	0.0335	961.5681
5	99	ORF16	SYYDYISR	0.0358	1065.5127
6	99	ORF16	YTPFYDDR	0.0419	1075.5027
7	< 1	ORF16	RIWDYDNELK	0.0204	1350.6766
8	99	ORF16	TQQLGGYQELTR	0.0375	1392.7377
9	99	ORF16	EPEGIYLYVNPR	0.0337	1448.7637
10	99	ORF16	YFDDIRPFLDEIYK	0.0285	1832.9266
11	99	ORF16	QNLLYGFFQTGEYER	0.0302	1846.8827
12	99	ORF16	QNLLYGFFQTGEYER	0.0238	1863.9027
13	99	ORF16	DNFREPEGIYLYVNPR	0.0129	1980.9827
14	< 1	ORF16	RQNLLYGFFQTGEYER	0.0002	2019.9807
15	99	ORF16	GIGDAGAWLQVSSGNVSGEVR	0.0168	2058.0298
16	99	ORF16	VYDYYLNDVSFEHGVENPR	0.0028	2315.0527
17	99	ORF16	ENTPDRPMQGIAVYGDDLYWLSGR	-0.0014	2752.2898

Fig. S2. Mass spectrometric analysis of two proteins from phage S24-1. (A) The structural proteins of phage S24-1. The structural proteins of phage S24-1 were separated using SDS-PAGE (7.5% gel) and visualized by CBB staining (see Fig. 2). The protein bands boxed in gray were digested by trypsin and analyzed by mass spectrometry. (B) Mass spectrometric analysis of the digested upper protein band. (C) Mass spectrometric analysis of the digested lower protein band. The mass spectra of the digested protein are shown on the left. The tables on the right show the mass spectrometry results. The peak numbers in the mass spectra correspond to those listed in the table. "Precursor molecular weight" is abbreviated as "Prec. MW."

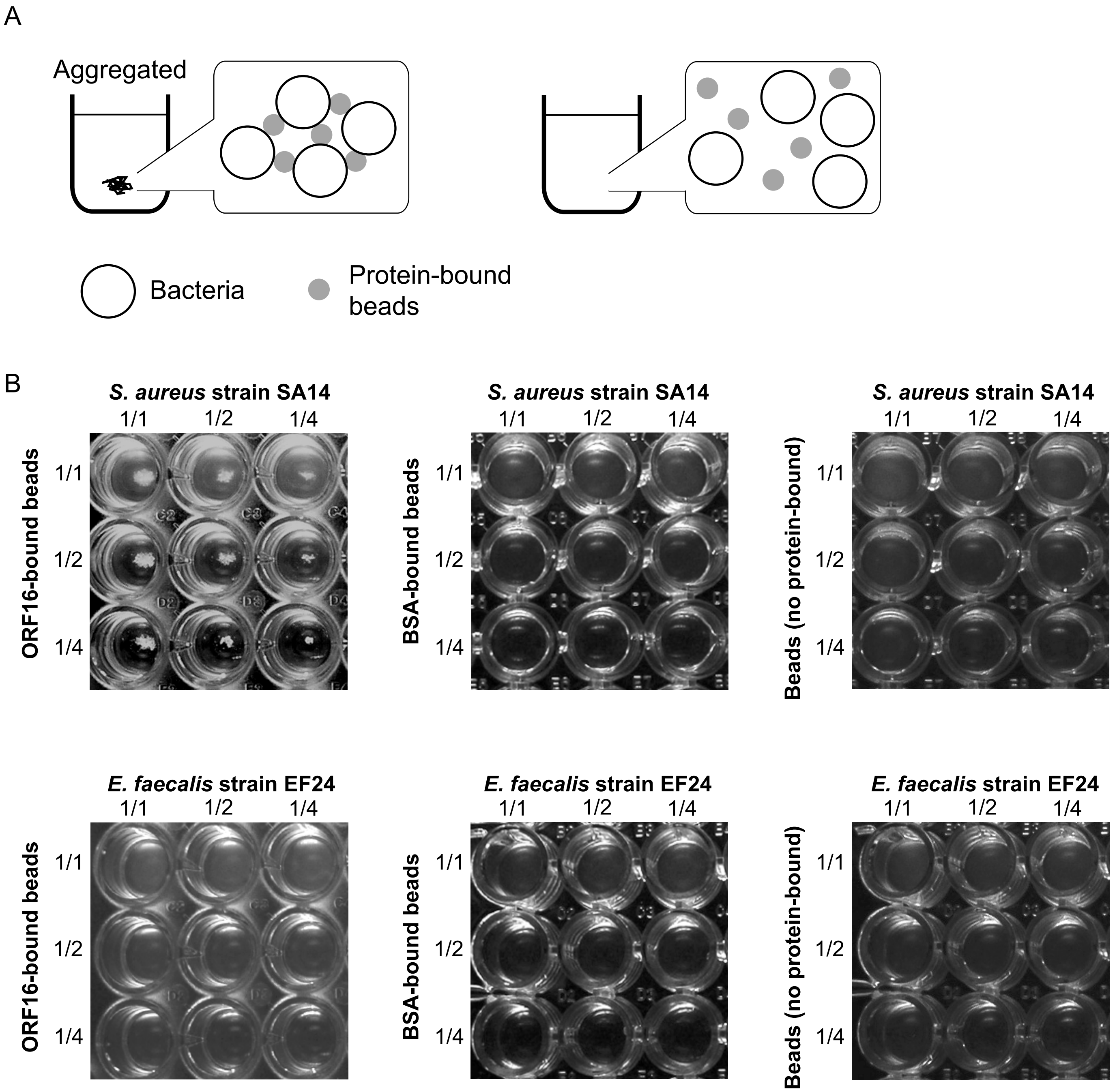

Fig. S3. Aggregation assay using rORF16-bound beads. (A) Schematic diagram of the aggregation assay system. If the protein had an affinity for the bacteria, aggregation with the bacteria was observed in the mixture (left). If the protein had no affinity for the bacteria, aggregation was not observed in the mixture (right). (B) Establishment of an aggregation assay system using rORF16-bound beads. rORF16-bound beads, BSA-bound beads, and beads only were prepared. Overnight cultures of *S. aureus* SA14 or *Enterococcus faecalis* EF24 were prepared. Twofold serial dilutions of the beads and bacteria were prepared using PBS. The beads were mixed with the bacteria and the aggregability was examined. Aggregation was only observed in the mixture of rORF16-bound beads with *S. aureus*, in a concentration-dependent manner.

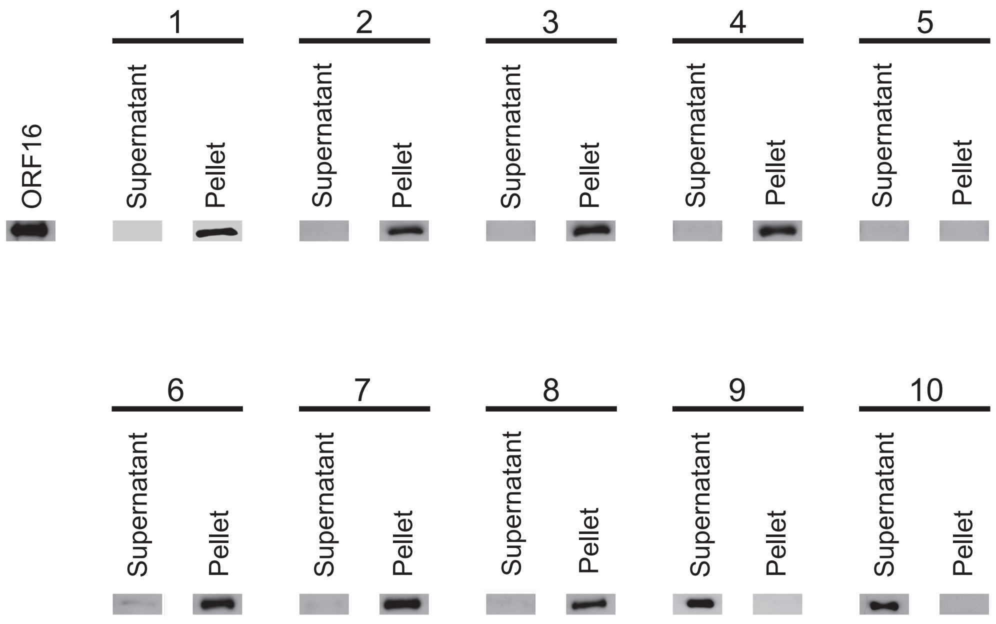

Fig. S4. Assessment of the rORF16 binding affinity with *S. aureus* samples treated with heat and various chemicals. After rORF16 was mixed with *S. aureus* samples treated with heat and various chemicals, the supernatants and bacterial cell pellets were separated by centrifugation. The samples were subjected to rORF16 detection by western blotting. The treatment group number is shown at the top for each result. The types of treatments and their designated numbers are as follows: untreated in No. 1, autoclaved in No. 2, SDS in No. 3, trichloroacetic acid (TCA) in No. 4, phenol–chloroform in No. 5, n-butanol in No. 6, Triton X-100 in No. 7, NaOH in No. 8, and HF in No. 9" has been replaced with "proteinase K in No. 5, phenol-chloroform in No. 6, n-butanol in No. 7, Triton X-100 in No. 8, NaOH in No. 9, and HF in No. 10. The result with ORF16 only is shown at the top left. Overall, the results agree with the aggregation assay results shown in Fig. 5. Unfortunately, *S. aureus* cells treated with proteinase K did not produce any bands from the pellet or the supernatant (data not shown), probably because proteinase K residuals appeared to digest the treated ORF16, even after thorough washing of the bacterial cells. The NaOH and HF-treated samples (Nos. 8 and 9) had no binding affinity with *S. aureus*.

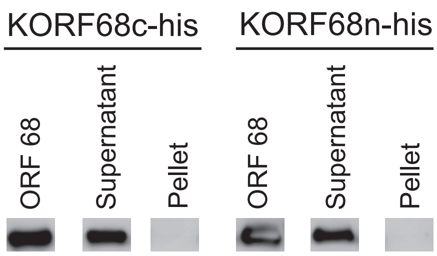

Fig. S5. Assessment of the binding activity using a recombinant ORF68 of phage K. The binding activity of phage K ORF68 was examined by western blotting using the recombinant proteins KORF68c-his (ORF68 with a 6 × His tag at the C-terminal) and KORF68n-his (ORF68 with a 6 × His tag at the N-terminal). *S. aureus* SA14 was sensitive to phage K infection. After incubation of the recombinant K_ORF68s with *S. aureus* strain SA14, the presence of the recombinant ORF68s in the solution or *S. aureus* cells was examined by western blotting using the anti-6 × His antibody. KORF68c-his and KORF68n-his had no binding activity with *S. aureus*, regardless of the presence of cationic ions such as $MgCl_2$ and $CaCl_2$.
