# Supplementary material for: In silico analysis of AHJD-like viruses, Staphylococcus aureus phages S24-1 and S13′, and study of phage S24-1 adsorption
Source: Microbiologyopen. 2014 Mar 4;3(2):257–70. doi: 10.1002/mbo3.166 (PMC3996573; doi:10.1002/mbo3.166)
Supplement: Table S1 — The phage lysis activities of phages S13′ and S24-1 against 89 S. aureus strains. [file mbo30003-0257-sd2.pdf]

Table S1. The phage lysis activities of phages S13' and S24-1 against 89 *S. aureus* strains.

S. aureus strain	Phages S24-1	Phages S13'	*S. aureus* strain	Phages S24-1	Phages S13'	*S. aureus* strain	Phages S24-1	Phages S13'
SA1	○	○	SA37	○	○	MR22	○	○
SA2	○	○	SA38	○	○	MR23	○	○
SA3	○	○	SA39	○	○	MR24	○	○
SA4	○	○	SA40	○	○	MR25	○	○
SA5	○	○	SA41	○	○	MR26	○	○
SA6	○	○	SA42	○	○	MR27	○	○
SA7	○	○	SA43	○	○	MR28	○	○
SA8	○	○	SA44	○	○	MR29	○	○
SA9	○	○	SA45	○	○	MR30	○	○
SA10	○	○	SA46	○	○			
SA12	○	○	SA47	○	○	TY12	○	○
SA14	○	○	SA48	○	○	TY21	○	×
SA15	○	○	MR1	○	○	TY115	○	○
SA16	○	○	MR3	○	○	TY116	○	×
SA17	○	○	MR4	○	○	TY117	○	○
SA18	○	○	MR5	○	○	TY127	○	○
SA19	○	×	MR6	○	○	TY147	○	○
SA21	○	○	MR7	○	○	TY148	○	○
SA22	○	○	MR8	○	×	TY154	○	○
SA23	○	○	MR9	○	○	TY162	○	○
SA24	○	○	MR11	○	○			
SA25	○	○	MR12	○	○	COL	○	○
SA26	○	×	MR13	○	○	RN4220	○	○
SA27	○	○	MR14	○	○	209P	○	○
SA28	○	○	MR15	○	○	NCTC10442	○	○
SA30	○	○	MR16	○	○	85/2082	○	○
SA31	○	×	MR17	○	○	N315	○	○
SA32	○	○	MR18	○	○	85/4547	○	○
SA33	○	○	MR19	○	○	MR108	○	○
SA34	○	○	MR20	○	○			
SA36	○	○	MR21	○	○			

×, not lysed; ○, lysis.

The phage lytic activity was examined using a spot test.

The *S. aureus* strains that were sensitive to phage S24-1, but insensitive to phage S13' are highlighted in gray.
