# Supplementary material for: In silico analysis of AHJD-like viruses, Staphylococcus aureus phages S24-1 and S13′, and study of phage S24-1 adsorption
Source: Microbiologyopen. 2014 Mar 4;3(2):257–70. doi: 10.1002/mbo3.166 (PMC3996573; doi:10.1002/mbo3.166)
Supplement: Table S2 — Bacterial strains used in this study and their phage S24-1 infectivity. [file mbo30003-0257-sd3.pdf]

Table S2. Bacterial strains used in this study and their phage S24-1 infectivity.

Bacteria			Phage S24-1 infectivity[a]
Genus	Species	Strain	
Bacillus	*cereus*	IID 1681 (ATCC 14579)	×
	subtilis	NCTC 3610	×
Escherichia	*coli*	DH5a	×
		BL21	×
Enterococcus	*faecalis*	EF24	×
		ND547 (ATCC49761)	×
		ATCC19433-U	×
	faecium	EFum2	×
		EFum3	×
Pseudomonas	*aeruginosa*	PAO1	×
Staphylococcus	*aureus* (methicillin-sensitive)	SA7	◯
		SA14	◯
		SA17	◯
		SA21	◯
		SA27	◯
		SA28	◯
		SA32	◯
		SA34	◯
		SA39	◯
		SA42	◯
	aureus (methicillin-resistant)	MRSA13	◯
		MRSA15	◯
		MRSA24	◯
		NCTC10442	◯
		85/2082	◯
		MR108	◯
	auricularis	GIFU10395	×
	epidermidis	SE17	×
		ATCC12228	×
	saprolyticus	ATCC15305	×

[a], "◯" and "×" indicate "plaque-forming" and "non-plaque-forming," respectively.
