# Supplementary material for: In silico analysis of AHJD-like viruses, Staphylococcus aureus phages S24-1 and S13′, and study of phage S24-1 adsorption
Source: Microbiologyopen. 2014 Mar 4;3(2):257–70. doi: 10.1002/mbo3.166 (PMC3996573; doi:10.1002/mbo3.166)
Supplement: Table S3 — List of primers used in this study. [file mbo30003-0257-sd4.pdf]

Table S3. List of primers used in this study.

Primer	Sequence
S24-1_ORF16F	CCCGGTACCATGGCATATAATGAAAACGATTTTAAATATTTTGACG
ORF16dF0	CGTAAAACGGTGTGTAACGTTCTC
ORF16dF1	AAAAGGTACCATAGATAAAATTATTCATGATGAATTTACTAAA
ORF16dF2	AAAAGGTACCAAATTAACGCCTAGTGGTGATTTA
ORF16dF3	AAAAGGTACCGAAGATGTAATTAATCACATTGACAAT
ORF16dF4	AAAAGGTACCTCTTTATTAATTGCATATACAACTGCT
ORF16dF5	AAAAGGTACCTTAGGTGGATATCAAGAATTAACAAG
S24-1_ORF16R	CCCAAGCTTTTTTTGATGTTTTGCTACCCAATCATACTC
ORF16dR0	CGTGAGTATGATTGGGTAGCAAAAC
ORF16dR1'	TTTTAAGCTTCCACTTACCAAATTCAAATTTTTGTG
ORF16dR2'	TTTTAAGCTTACCTTTTAACGTTAATGCAACAAAAC
ORF16dR3'	TTTTAAGCTTAACTGCAATACCTTGCATAGG
ORF16dR4'	TTTTAAGCTTCAACCATATTTTTATCTCACCGTT
ORF16dR5'	TTTTAAGCTTTTCTGCTTTAAAGTCATTCATTTGTTT
K_ORF68_F	TTTGGTACCATGGCATTAAATTTTACTACAATAACGGAAAAC
K_ORF68_R	TTTAAGCTTCTATGGCATATTAATACCTATAATTCTTGTAAC
K_ORF68_HIS_R	TTTAAGCTTATGATGATGATGATGATGTGGCATATTAATACCTATAATTCTTGTAACGTA
