# Supplementary material for: In silico analysis of AHJD-like viruses, Staphylococcus aureus phages S24-1 and S13′, and study of phage S24-1 adsorption
Source: Microbiologyopen. 2014 Mar 4;3(2):257–70. doi: 10.1002/mbo3.166 (PMC3996573; doi:10.1002/mbo3.166)
Supplement: Table S4 — PCR products and primer lists. [file mbo30003-0257-sd5.pdf]

Table S4. PCR products and primer lists.

Primers	PCR method	Description of PCR product	Recombinant proteins	Location of 6 × His tag[a] (expression vector)	Description of recombinant protein
S24-1_ORF16F<br>S24-1_ORF16R	PCR with genomic DNA	*orf16* cloned into pUC18 (pUC18	rORF16	N (pCold II)	Recombinant ORF16
ORF16dF0<br>ORF16dF1	inverse PCR with pUC18 *orf16*	5'-truncated *orf16*	ORF16F1	N (pCold II)	N-terminally deleted recombinant ORF16
ORF16dF0<br>ORF16dF2	inverse PCR with pUC18 *orf16*	5'-truncated *orf16*	ORF16F2	N (pCold II)	N-terminally deleted recombinant ORF16
ORF16dF0<br>ORF16dF3	inverse PCR with pUC18 *orf16*	5'-truncated *orf16*	ORF16F3	N (pCold II)	N-terminally deleted recombinant ORF16
ORF16dF0<br>ORF16dF4	inverse PCR with pUC18 *orf16*	5'-truncated *orf16*	ORF16F4	N (pCold II)	N-terminally deleted recombinant ORF16
ORF16dF0<br>ORF16dF5	inverse PCR with pUC18 *orf16*	5'-truncated *orf16*	ORF16F5	N (pCold II)	N-terminally deleted recombinant ORF16
ORF16dR0<br>ORR16dR1	inverse PCR with pUC18 *orf16*	3'-truncated *orf16*	ORF16R1	N (pCold II)	C-terminally deleted recombinant ORF16
ORR16dR0<br>ORR16dR2	inverse PCR with pUC18 *orf16*	3'-truncated *orf16*	ORF16R2	N (pCold II)	C-terminally deleted recombinant ORF16
ORR16dR0<br>ORR16dR3	inverse PCR with pUC18 *orf16*	3'-truncated *orf16*	ORF16R3	N (pCold II)	C-terminally deleted recombinant ORF16
ORR16dR0<br>ORR16dR4	inverse PCR with pUC18 *orf16*	3'-truncated *orf16*	ORF16R4	N (pCold II)	C-terminally deleted recombinant ORF16
ORR16dR0<br>ORR16dR5	inverse PCR with pUC18 *orf16*	3'-truncated *orf16*	ORF16R5	N (pCold II)	C-terminally deleted recombinant ORF16
K_ORF68_F<br>K_ORF68_R	PCR with genomic DNA	*orf68* of phage K	KORF68n-his	N (pCold II)	Recombinant ORF68 of phage K
K_ORF68_F<br>K_ORF68_HIS_R	PCR with genomic DNA	*orf68* of phage K	KORF68c-his	C (pCold III)	Recombinant ORF68 of phage K

[a], "N" and "C" indicate "N-terminal" and "C-terminal," respectively.
