# Supplementary material for: In silico analysis of AHJD-like viruses, Staphylococcus aureus phages S24-1 and S13′, and study of phage S24-1 adsorption
Source: Microbiologyopen. 2014 Mar 4;3(2):257–70. doi: 10.1002/mbo3.166 (PMC3996573; doi:10.1002/mbo3.166)
Supplement: Table S6 — Annotation of the phage S24-1 genome. [file mbo30003-0257-sd7.pdf]

Table S6. Annotation of the phage S24-1 genome.

ORF	Location		Orientation	Length		RBS[a]	MW (kDa)	Predicted ORF function
	Start	End		(nt)	(AA)			
**1**	377	559	+	183	60	CAAGGAGGTAACAAA	6.9	
**2**	552	854	+	303	100	TATGGAGTGATAACA	11.6	
**3**	872	1108	+	237	78	AGGAGTGATATAATA	9.2	
**4**	1132	1494	+	363	120	AGAGGAGAAATAAAA	14.1	Single stranded DNA binding protein
**5**	1550	1726	+	177	58	TTATGAGGTGCTTAA	7.2	
**6**	1728	2132	+	405	134	TTAAGGAGATATAAA	16.1	
**7**	2125	2295	+	171	56	TTTAGGAAAAGTGATTGACC	6.5	
**8**	2298	2780	+	483	160	TGGAGGTCGTTAGCA	19.5	
**9**	2828	4075	+	1248	415	ACAAGGAGAAAAAAC	50.4	
**10**	4090	6375	+	2286	761	ATATAAAGGTGTGTAAGATT	90.4	DNA polymerase
**11**	6490	7929	-	1440	479	TTCTGAAAAGAGTGATAATA	52.3	Tail lysin
**12**	7904	8326	-	423	140	TTTAGAGCAGGTGTATAAAA	16.2	Holin
**13**	8329	10092	-	1764	587	TTTATGGAGGTAAAA	68.4	Major tail protein
**14**	10148	11593	-	1446	481	TAAGAGGTGTGAACA	55.3	Minor tail protein
**15**	11656	12408	-	753	250	ATAGGAGTGATATAA	28.6	Endolysin
**16**	12420	14348	-	1929	642	TTTGGTAAAGGTGGAAAATT	74.5	Minor structural protein
**17**	14361	15116	-	756	251	TGACGAAAGTAGTGAATACA	29.2	Lower collar protein
**18**	15109	16092	-	984	327	ATAGAGGTGCTATAA	37.8	Upper collar protein
**19**	16107	17318	-	1212	403	TTGAGGAGGAATAATAAATC	46.1	Major capsid protein
**20**	17325	17507	-	183	60	AGATTAGGAGGTACTTAAAC	6.9	
**21**	17521	17880	-	360	119	TTTGGAGGTGTCAAA	13.8	

[a], Putative ribosome-binding site in red.
